# Comparative Chloroplast and Mitochondrial Genomics of *Sorbus* L. Sensu Lato: Genome Diversity, Structural Variation, and Phylogeny

**DOI:** 10.3390/ijms27209324

**Published:** 2026-10-20

**Authors:** Yuxia Wu, Wenhua Yang, Yongqi Zheng, Xuedan Yu, Chuanhong Zhang

**Affiliations:** State Key Laboratory of Tree Genetics and Breeding, Research Institute of Forestry, Chinese Academy of Forestry, Beijing 100091, China; wuyux3@163.com (Y.W.);

**Keywords:** organelle genome evolution, genetic diversity, structural variation, phylogeny, Rosaceae

## Abstract

*Sorbus* L. sensu lato represents a taxonomically complex group within Rosaceae, but organelle genome variation remains insufficiently characterized, particularly at the intraspecific level. Here, we assembled the complete mitochondrial genome of *S. discolor* and 214 complete chloroplast genomes, comprising 71 *S. discolor* and 143 *S. pohuashanensis* accessions, and performed comparative and phylogenetic analyses alongside published genomes. The *S. discolor* mitogenome spanned 372,987 bp and contained 56 genes, representing the smallest genome yet exhibiting the highest number of annotated genes among the six mitogenomes examined; it also possessed the fewest simple and long sequence repeats. A GAA-to-TAA substitution introduced a premature stop codon in mitochondrial *sdh3*, resulting in pseudogenization. Across six species, *sdh3* was either intact, pseudogenized, or absent, whereas putative nuclear *sdh3* homologs were identified in *S. discolor* and *S. pohuashanensis*. Comparative analysis of 263 chloroplast genomes revealed that highly variable regions differed between interspecific and intraspecific datasets, whereas *trnH-GUG–psbA* consistently exhibited relatively high nucleotide diversity. The chloroplast phylogeny placed all sampled compound-leaved species within *Sorbus* s. s., whereas the sampled simple-leaved taxa were positioned closer to the base, with a similar pattern observed in the mitochondrial phylogeny based on six species. These findings reveal contrasting patterns of organelle genome variation and provide resources for genetic diversity assessment, marker development, and future studies of *Sorbus* genetic resources.

## 1. Introduction

*Sorbus* L. sensu lato (*Sorbus* s. l., hereafter abbreviated as *Sorbus*) comprises approximately 116 species distributed across temperate regions of the Northern Hemisphere, including Asia, Europe, and North America (USDA-ARS Germplasm Resources Information Network, https://www.ars-grin.gov/ (accessed on 19 September 2026)). China is a major center of diversity for *Sorbus*, hosting approximately 70 species [1]. *Sorbus* species are multifunctional trees with ornamental, edible [2,3], medicinal [4], and timber values.

The taxonomic circumscription of *Sorbus* has long been controversial. Linnaeus initially recognized only two pinnate-leaved species [5], whereas later taxonomists proposed either a narrow concept restricted to pinnate-leaved species [6,7,8], or a broad concept comprising both simple- and pinnate-leaved groups [9,10,11]. Recent molecular phylogenetic studies indicate that *Sorbus* is not monophyletic and comprises several distinct lineages, including *Aria*, *Chamaemespilus*, *Micromeles*, *Torminalis*, *Cormus*, and *Sorbus* s. s. [12]. Hybridization, polyploidization, and apomixis are major contributors to this taxonomic complexity and have played important roles in the ongoing evolution of *Sorbus* [13,14,15,16]. Hybridization is often associated with variation in morphological traits, further complicating the correspondence between morphology and evolutionary relationships [17]. Consequently, resolving the evolutionary relationships among *Sorbus* lineages requires additional genomic evidence.

Organelle genomes are widely used to investigate evolutionary relationships and genomic variation in plants [18,19]. Chloroplast genomes of most angiosperms exhibit a relatively conserved quadripartite structure, limited recombination, and moderate rates of nucleotide substitution [19]. Compared with a few plastid markers, whole-chloroplast genome data provide substantially more sequence information and allow for a more detailed assessment of genetic variation [18]. With the increasing availability of complete chloroplast genomes and resequencing data, pan-plastome analyses have become increasingly valuable for investigating variation among individuals and at the intraspecific level [20,21,22,23,24]. Studies in several plants, such as *Forsythia suspensa*, have demonstrated that dense chloroplast genome sampling can reveal substantial intraspecific variation and genetic differentiation [20]. In contrast to the relatively conserved chloroplast genomes, plant mitochondrial genomes vary greatly in size and structure and often undergo extensive rearrangement [25]. Large amounts of non-coding and repetitive sequences contribute to the complexity and diversity of plant mitochondrial genomes, and repetitive sequences may promote homologous recombination and structural rearrangements [26,27]. Similar patterns of genome-size variation, repeat-associated rearrangements, and structural complexity have recently been reported in *Rosa* [28], *Camellia* [29,30,31], *Hippophae* [32], *Neocinnamomum* [33], and *Glycyrrhiza* [34]. Thus, chloroplast and mitochondrial genomes provide complementary perspectives on sequence variation, genome structural evolution, and maternal evolutionary relationships in plants.

Complete chloroplast genomes have been widely applied to phylogenetic and taxonomic studies of *Sorbus* [35,36,37]. Ulaszewski et al. [35] assembled the chloroplast genome of *S. aucuparia* and compared it with those of *S. aria*, *S. chamaemespilus*, and *S. torminalis*. Their phylogenetic analyses supported the separation of *Aria*, *Chamaemespilus*, and *Torminalis* from *Sorbus* s. s. Tang et al. [36] subsequently analyzed 29 chloroplast genomes of *Sorbus* s. s., including 16 newly sequenced genomes representing two subgenera and eight sections. They identified several hypervariable regions and repeat sequences with potential value as molecular markers and further evaluated the infrageneric classification of *Sorbus* s. s., supporting two major subgeneric lineages while revealing inconsistencies with the existing sectional classification. Zhang and Ling [37] characterized the chloroplast genomes of eight *Sorbus* s. s. species and compared them with 12 previously published genomes. Their analyses confirmed the high conservation of plastome structure and gene order, identified four highly variable intergenic regions, and further investigated phylogenetic relationships within *Sorbus* s. s. However, most previous studies have focused on interspecific comparisons with limited sampling within individual species, leaving intraspecific plastome variation and diversity poorly understood. Mitochondrial genomic resources for *Sorbus* are even more limited, with complete mitochondrial genomes previously available in NCBI for only five *Sorbus* species. Consequently, comparative analyses of mitochondrial genome variation across the genus remain constrained. Overall, intraspecific plastome variation and broader comparative mitochondrial genomics remain insufficiently studied in *Sorbus*.

In this study, we analyzed 263 complete chloroplast genomes of *Sorbus*, including 71 newly assembled genomes of *S. discolor* and 143 newly assembled genomes of *S. pohuashanensis*, together with six representative mitochondrial genomes, one of which was newly assembled for *S. discolor*. We focused on chloroplast genome variation at both interspecific and intraspecific levels, genetic diversity in the two densely sampled species, mitochondrial structural variation, and phylogenetic relationships inferred from both organelle genomes. By integrating extensive intraspecific plastome sampling with comparative mitochondrial genomics, this study provides a broader understanding of organelle genome variation and evolutionary patterns in *Sorbus*.

## 2. Results

### 2.1. General Features of Chloroplast and Mitochondrial Genomes

Assembly quality assessment showed consistently high coverage across all 214 newly assembled plastomes. The GetOrganelle average base coverage ranged from 202.6× to 586.0× (mean: 473.30×). Read-mapping depth ranged from 483.57× to 13,533.80× (mean: 2763.19×), and genome coverage ranged from 99.9906% to 100%. Among the 214 plastomes, 194 showed 100% coverage. All final plastomes were complete, gap-free, and contained no ambiguous bases. The *Sorbus* pan-chloroplast genomes comprised 263 complete chloroplast genomes, all exhibiting the typical quadripartite structure consisting of a LSC region, a SSC region, and a pair of inverted-repeat regions (IRa and IRb). The total length of pan-chloroplast genomes ranged from 159,341 to 160,609 bp, with the LSC, SSC, and IR regions spanning 87,351–88,478 bp, 18,761–19,387 bp, and 26,357–26,416 bp, respectively. The GC content ranged from 36.42% to 36.66%, with IR regions showing the highest values (42.61–42.69%) and SSC regions having the lowest GC content (30.19–30.53%). At the intraspecific level, the chloroplast genome sizes of *S. discolor* and *S. pohuashanensis* ranged from 160,005–160,197 bp and 159,869–160,206 bp, respectively. All newly assembled 71 *S. discolor* and 143 *S. pohuashanensis* chloroplast genomes consistently contained 112 unique genes, including 78 protein-coding genes, 30 tRNA genes, and four rRNA genes (Table 1, Supplementary Table S1). Apparent differences in gene number among the additional published plastomes were not interpreted as biological gene gain or loss because they may be influenced by differences in annotation status, particularly the treatment of partial and pseudogene copies.

The newly assembled *S. discolor* mitochondrial genome spans 372,987 bp with a GC content of 45.36%. This assembly is fully supported by PacBio HiFi reads, achieving 100% genome coverage and a mean read depth of 340.04×. Comparative analysis of the six mitochondrial genomes revealed substantially greater variation in genome size than observed in the chloroplast dataset. Genome sizes ranged from 372,987 bp in *S. discolor* to 455,361 bp in *S. alnifolia*. In contrast, mitochondrial GC content was highly conserved, varying only from 45.20% to 45.39%. The total number of unique genes ranged from 53 to 56, comprising 33–34 protein-coding genes, 17–19 tRNA genes, and 2–4 rRNA genes (Table 2).

### 2.2. Conserved Chloroplast Genome Junctions and Mitochondrial Structural Variation

**Figure 1:**
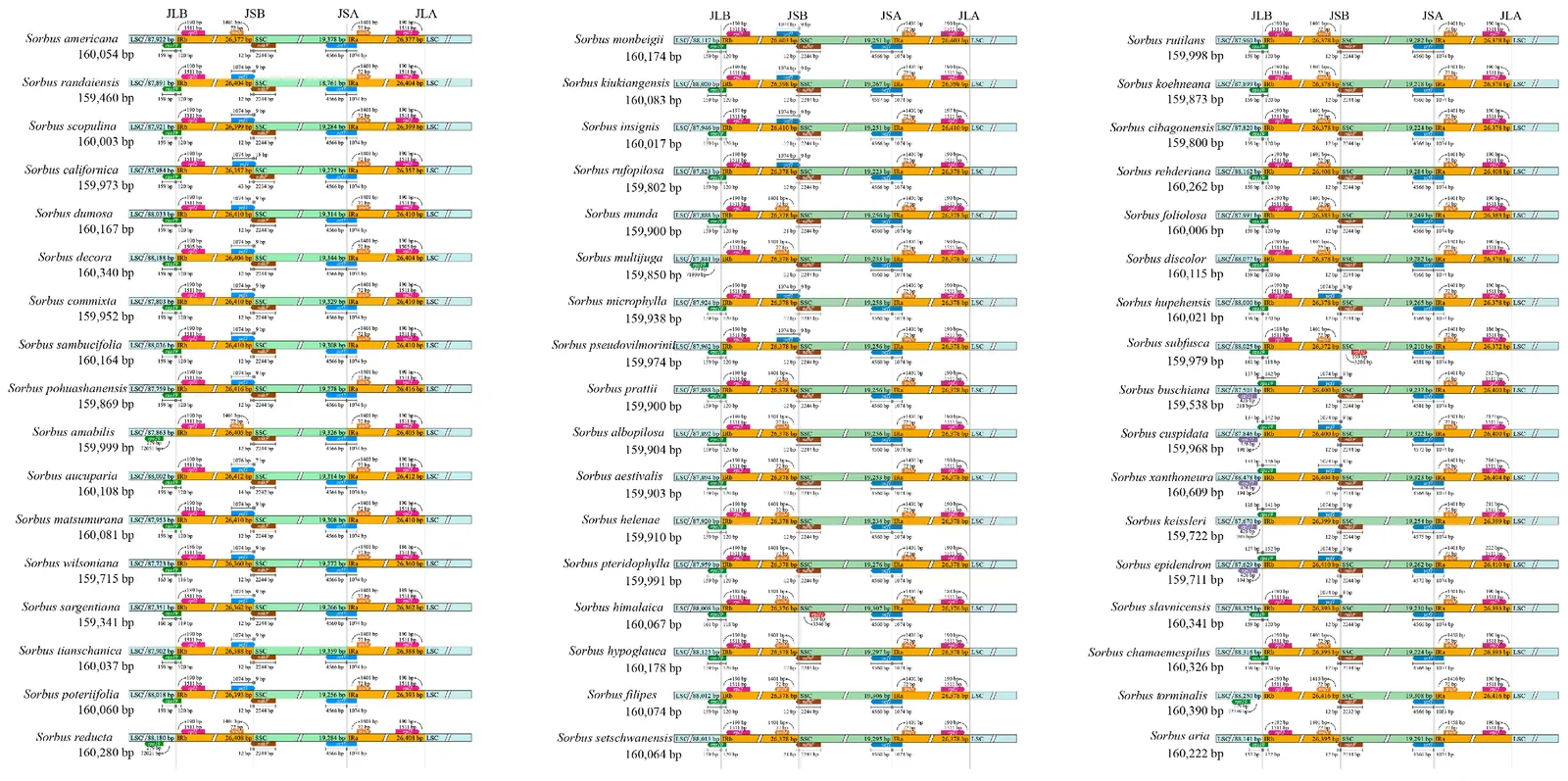
Comparative structural evolution of chloroplast genomes in *Sorbus*.

Comparative analysis of the LSC/IR and SSC/IR junctions across the 51 *Sorbus* sensu lato chloroplast genomes revealed that boundary regions were generally conserved, with only minor interspecific variations (Figure 1). The IR regions ranged from 26,357 to 26,416 bp. At the LSC–IRb junction (JLB), *rps19* typically spanned the boundary, although the lengths of the fragments located in the LSC and IRb varied slightly among species. A different pattern was observed in five species (*S. buschiana*, *S. cuspidata*, *S. xanthoneura*, *S. keissleri*, and *S. epidendron*), in which *rpl22* was located near the JLB but remained entirely within the LSC. In *S. multijuga* and *S. torminalis*, *rps19* was located entirely within the LSC. The *rpl2* gene was situated within the IR region in all chloroplast genomes.

At the IRb–SSC junction (JSB), ψ*ycf1* crossed the boundary in most species, with approximately 1074 bp located in the IRb and a short 9-bp fragment extending into the SSC. The *ndhF* gene was located in the SSC and was typically 12–21 bp from the JSB. In *S. himalaica* and *S. subfusca*, *rpl32* was the closest annotated gene to JSB and was 3346 and 3286 bp from the boundary, respectively. At the SSC–IRa junction (JSA), the intact *ycf1* gene crossed the boundary in all species, with the majority of the gene located in the SSC and about 1.07–1.08 kb extending into IRa. The duplicated 5′ end of *ycf1* in IRb formed the truncated ψ*ycf1* copy near JSB. The *trnN*-*GUU* gene was completely located within IRa. At the IRa–LSC junction (JLA), *rpl2* remained within IRa, with only minor variations in its distance from the boundary among species. The IR/SC boundaries were highly conserved across the 51 chloroplast genomes, and the observed differences were mainly caused by small shifts in junction positions rather than large-scale IR expansions or contractions.

**Figure 2:**
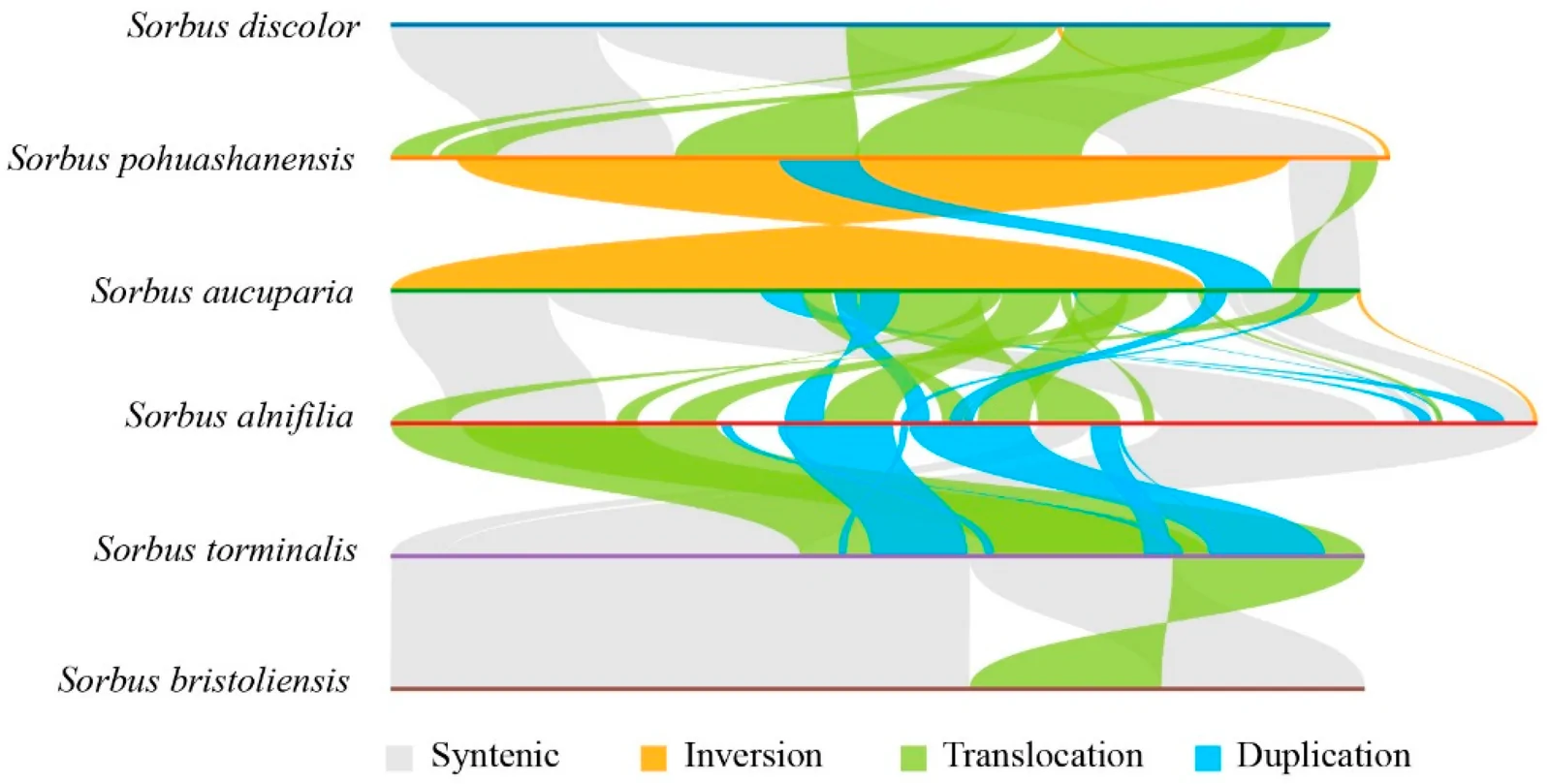
Comparative structural evolution of mitochondrial genomes in *Sorbus*.

In contrast, comparisons of the six mitochondrial genome assemblies revealed substantial structural differences. Although large homologous syntenic blocks were retained among the six species, their order and orientation differed substantially. A region classified as an inversion was observed between *S. pohuashanensis* and *S. aucuparia*, whereas multiple regions classified as translocations and duplications were distributed across comparisons involving *S. aucuparia*, *S. alnifolia*, and other representative species. *S. torminalis* and *S. bristoliensis* retained comparatively extensive syntenic regions, although translocated blocks were also present (Figure 2).

### 2.3. Repeat Sequence Variation in Chloroplast and Mitochondrial Genomes

**Figure 3:**
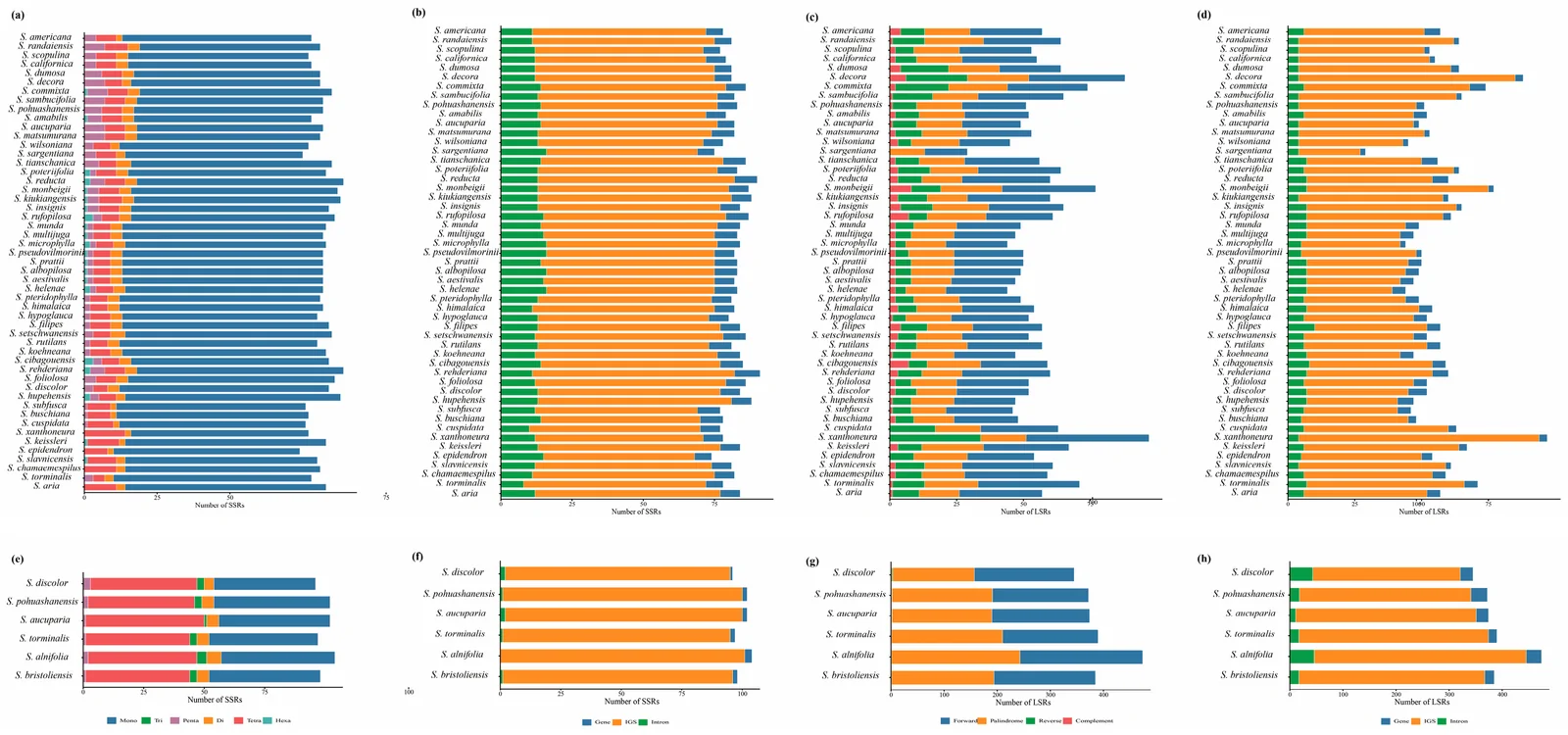
Distribution and composition of repetitive sequences in the chloroplast and mitochondrial genomes of *Sorbus*. (**a**) Composition of chloroplast SSRs by motif type. (**b**) Distribution of chloroplast SSRs across genomic regions. (**c**) Composition of chloroplast LSRs by repeat type. (**d**) Distribution of chloroplast LSRs across genomic regions. (**e**) Composition of mitochondrial SSRs by motif type. (**f**) Distribution of mitochondrial SSRs across genomic regions. (**g**) Composition of mitochondrial LSRs by repeat type. (**h**) Distribution of mitochondrial LSRs across genomic regions.

Each of the 263 chloroplast genomes contained 73–91 SSRs. Mononucleotide repeats were the most abundant type (58–75), followed by tetranucleotide (4–14), dinucleotide (2–5), pentanucleotide (0–8), and hexanucleotide repeats (0–3), whereas no trinucleotide repeats were detected. Mononucleotide SSRs were dominated by T motifs (31–41), followed by A motifs (24–30). Most SSRs were located in intergenic regions (53–71), with fewer found in genes (6–9) and introns (8–16) (Figure 3a,b, Supplementary Table S2). Specifically, 82–91 SSRs were detected in 71 accessions of *S. discolor*, whereas 73–87 SSRs were identified in 143 accessions of *S. pohuashanensis*. For long sequence repeats (LSRs), 29–97 repeats were detected in each chloroplast genome. Forward (16–46) and palindromic repeats (13–23) were more common than reverse (0–34) and complement repeats (0–8). Most LSRs were also distributed in intergenic regions (23–90), while fewer were found in genes (2–7) and introns (4–10). The number of LSRs per chloroplast genome ranged from 46 to 56 across the 71 *S. discolor* accessions and from 44 to 84 across the 143 *S. pohuashanensis* accessions (Figure 3c,d, Supplementary Table S3).

SSRs were also detected in all six mitochondrial genomes, with numbers varying among species (Figure 3e,f, Supplementary Table S4). Mononucleotide repeats were the most abundant SSR type and mitochondrial SSRs were primarily distributed in intergenic regions, with fewer located in genes and introns. The number of LSRs also varied among the six mitochondrial genomes, with the highest count detected in *S. alnifolia* and the lowest in *S. discolor* (Figure 3g, Supplementary Table S5). Forward and palindromic repeats were the predominant types, whereas reverse and complement repeats were less common or absent in some species. Most mitochondrial LSRs were located in intergenic regions (Figure 3h).

### 2.4. Mitochondrial sdh3 Variation and Putative Nuclear Homologs

**Figure 4:**
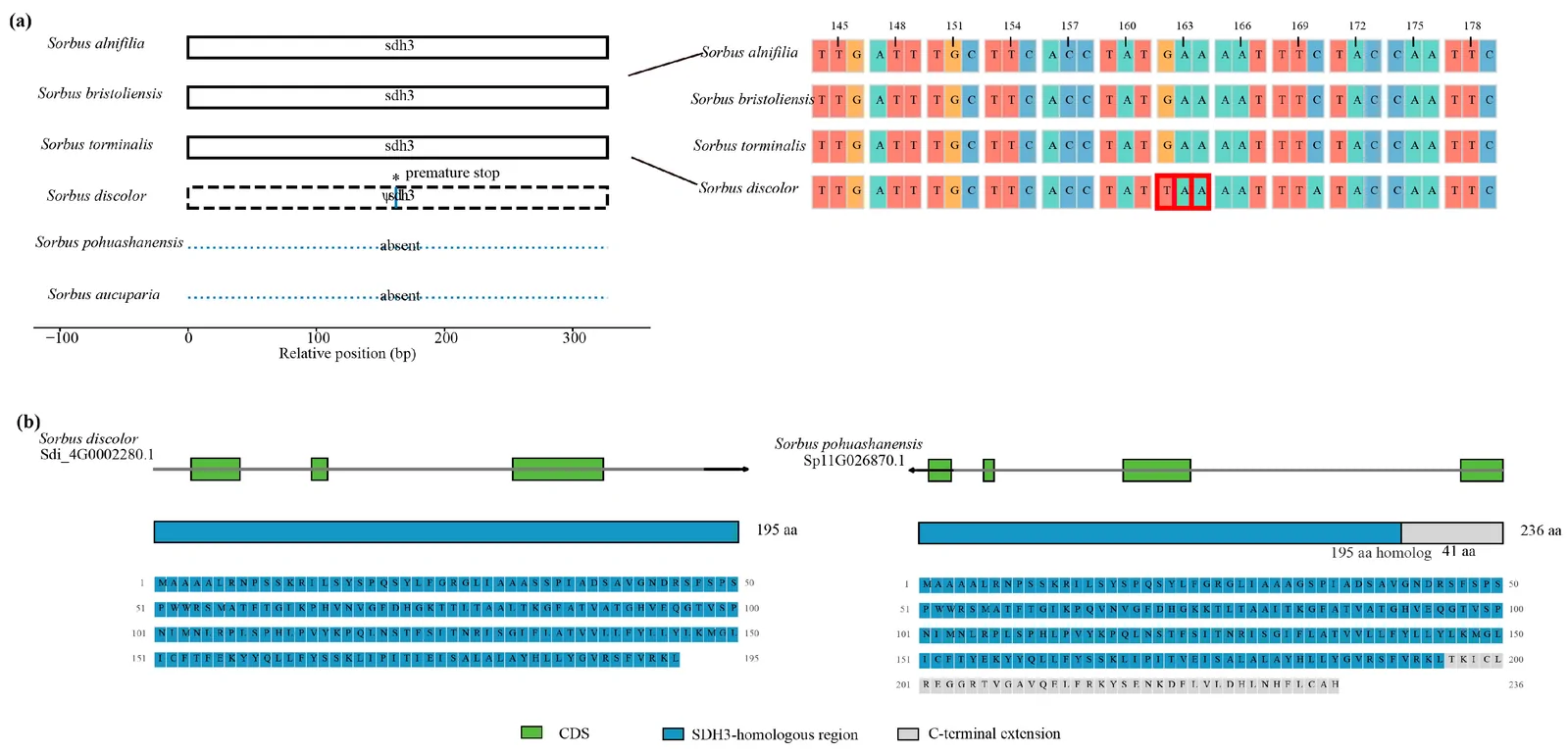
Mitochondrial *sdh3* degeneration and putative nuclear *sdh3* homologs in *Sorbus*. (**a**) Variation in mitochondrial *sdh3* status among six representative species. The asterisk indicates the premature stop codon in *S. discolor*, and the red box highlights the GAA-to-TAA substitution resulting in premature termination. (**b**) Gene structures and protein sequences of putative nuclear *sdh3* homologs in *S. discolor* and *S. pohuashanensis*. CDS structures are displayed in the 5′–3′ transcriptional orientation.

Comparative analysis of mitochondrial protein-coding gene content revealed variation in mitochondrial *sdh3* among the six examined species. Intact 327-bp *sdh3* coding sequences were present in *S. alnifolia*, *S. bristoliensis*, and *S. torminalis*. In *S. discolor*, a 327-bp region homologous to *sdh3* was retained, but its open reading frame was disrupted by a premature stop codon. At the corresponding site, the GAA codon encoding glutamic acid in the three intact sequences was replaced by TAA in *S. discolor*, resulting in premature termination at approximately amino-acid position 55 and indicating pseudogenization. No intact mitochondrial *sdh3* homolog was detected in *S. pohuashanensis* or *S. aucuparia* (Figure 4a). 

Putative *sdh3* homologs were also identified in the nuclear genomes of *S. discolor* and *S. pohuashanensis*. In the nuclear genome of *S. discolor*, *Sdi_4G0002280.1* was identified as the candidate *sdh3* homolog. This gene contains three CDS exons and encodes a predicted 195-aa protein. In the nuclear genome of *S. pohuashanensis*, *Sp11G026870.1* was identified as the corresponding candidate, containing four CDS exons and encoding a predicted 236-aa protein (Figure 4b). BLASTp searches of the predicted protein sequences against the NCBI protein database revealed that the 195-aa protein encoded by *Sdi_4G0002280.1* shared 100% query coverage and 94% amino acid identity with the 195-aa mitochondrial-like SDH3-1 protein of *Malus sylvestris* (Supplementary Table S6). The first 195 aa of the protein encoded by *Sp11G026870.1* showed 96% amino-acid identity with the complete 195-aa mitochondrial-like SDH3 protein of *Pyrus communis* (Supplementary Table S7), while the remaining 41 aa constituted an additional C-terminal extension. These results support the presence of putative nuclear *sdh3* homologs in both *S. discolor* and *S. pohuashanensis* despite the different states of their mitochondrial *sdh3* copies.

### 2.5. Plastome Sequence Diversity and Regional Nucleotide-Diversity Variation

The alignment of the 263 plastomes comprised 145,923 positions, with 126,230 sites retained after excluding those containing gaps or missing data. Across the sampled *Sorbus* taxa, 8560 segregating sites (S) and 8730 mutations (Eta) were detected, defining 99 haplotypes. Genome-wide sequence diversity in this mixed-taxon dataset was characterized by a haplotype diversity (Hd) of 0.970 and nucleotide diversity (Pi) of 0.00130 (Table 3). 

At the intraspecific level, analysis of the 71 *S. discolor* plastomes revealed 316 segregating sites and 320 mutations, defining 19 haplotypes (Hd = 0.873, Pi = 0.00025, and k = 32.963). Among the 143 *S. pohuashanensis* plastomes, 359 segregating sites and 365 mutations were identified, defining 44 haplotypes (Hd = 0.959, Pi = 0.00049, and k = 64.378). Consequently, *S. pohuashanensis* exhibited approximately 1.96-fold higher nucleotide diversity than *S. discolor*, along with higher Hd and k values. Geographic mapping indicated that plastome haplotypes were heterogeneously distributed among sampling localities for both *S. discolor* and *S. pohuashanensis* (Figure S1).

**Figure 5:**
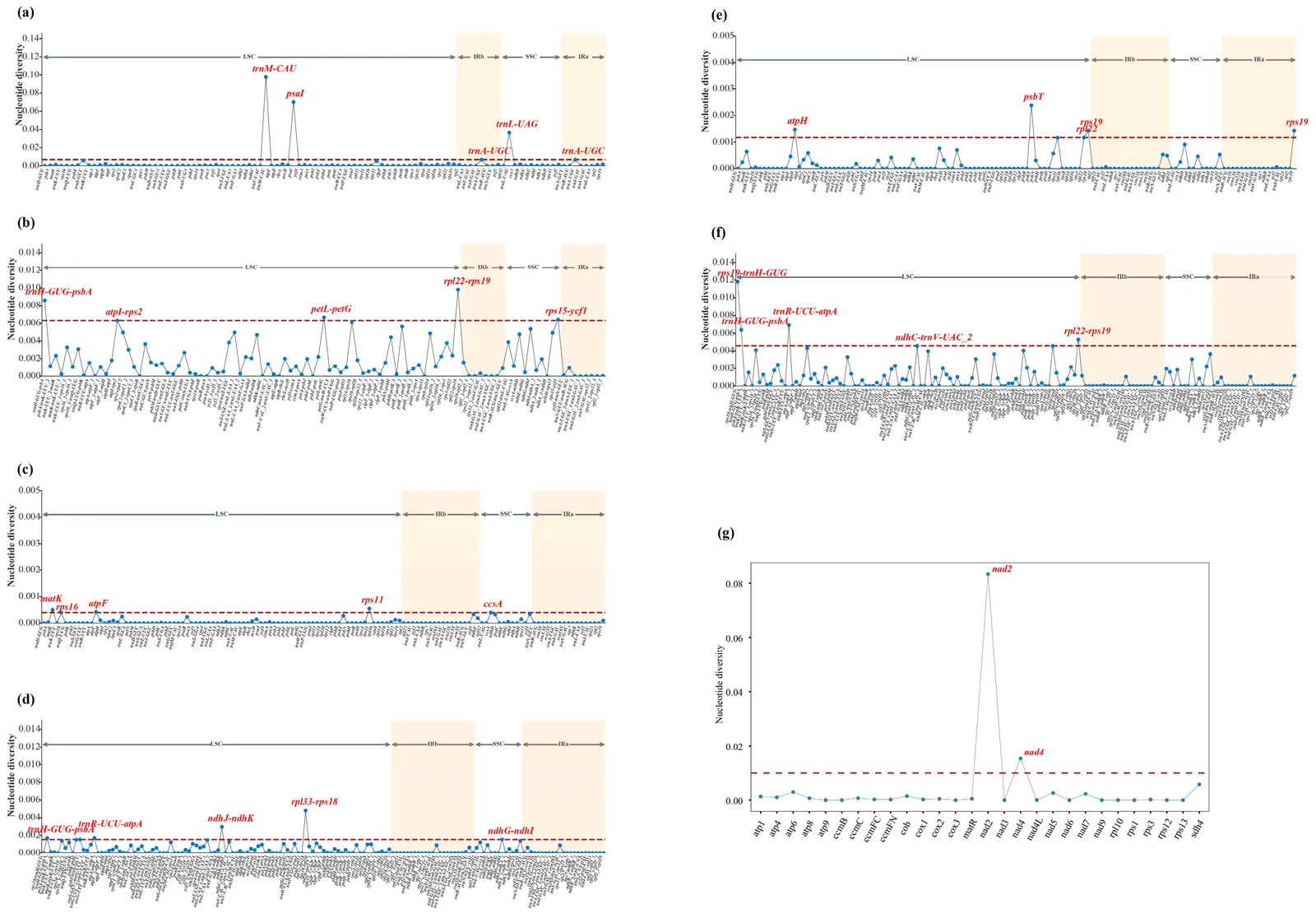
Nucleotide diversity across chloroplast and mitochondrial genomes of *Sorbus*. (**a**,**b**) Pi values of shared gene and intergenic regions, respectively, across the complete dataset of sampled *Sorbus* taxa (*n* = 263). (**c**,**d**) Corresponding profiles for *S. discolor* (*n* = 71). (**e**,**f**) Corresponding profiles for *S. pohuashanensis* (*n* = 143). (**g**) Nucleotide diversity among homologous mitochondrial gene regions of the six representative species. Red dashed lines indicate the thresholds used to highlight highly variable regions.

Nucleotide diversity of shared genes and intergenic regions varied across the chloroplast genomes. In the complete dataset of sampled *Sorbus* taxa, *trnM-CAU* exhibited the highest Pi value among the shared genes, followed by *psaI*, *trnL-UAG* and *trnA-UGC* (Figure 5a). Among the intergenic regions, relatively high Pi values were detected in *trnH-GUG–psbA*, *atpI–rps2*, *petL–petG*, *rpl22–rps19*, and *rps15–ycf1* (Figure 5b). In *S. discolor*, several genes, including *matK*, *rps16*, *atpF*, *rps11*, and *ccsA*, displayed relatively high Pi values (Figure 5c), whereas the main variable intergenic regions included *trnH-GUG–psbA*, *trnR-UCU–atpA*, *ndhJ–ndhK*, *rpl33–rps18*, and *ndhG–ndhI* (Figure 5d). In *S. pohuashanensis*, *psbT* showed the highest Pi value among shared genes, with relatively high values also detected in *atpH* and *rps19* (Figure 5e). Several intergenic regions exhibited high variation, including *rps19–trnH-GUG*, *trnH-GUG–psbA*, *trnR-UCU–atpA*, *ndhC–trnV-UAC*, and *rpl22–rps19* (Figure 5f). Among the 28 shared mitochondrial CDSs, Pi values ranged from 0 to 0.08333. The highest nucleotide diversity was detected in *nad2* (Pi = 0.08333), followed by *nad4* (Pi = 0.01534). 

### 2.6. Codon-Usage Patterns, Predicted Mitochondrial RNA Editing and Selective Pressure Analysis

**Figure 6:**
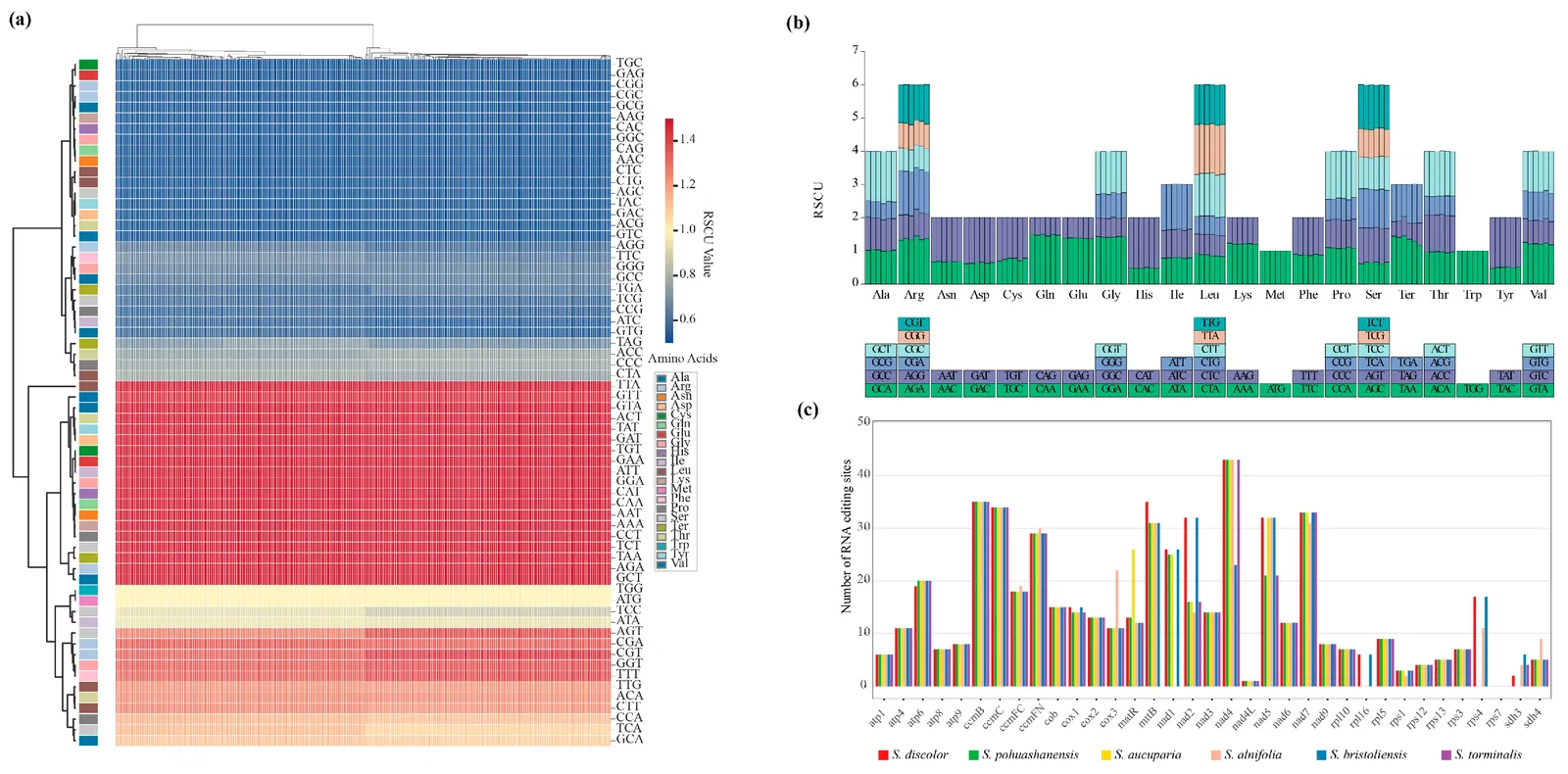
Codon-usage patterns and predicted mitochondrial RNA-editing sites in *Sorbus*. (**a**) Heatmap of RSCU across the 263 chloroplast genomes. (**b**) RSCU profiles of the six representative mitochondrial genomes, grouped by amino acid. (**c**) Number of predicted RNA-editing sites in mitochondrial protein-coding genes of the six representative species.

To characterize codon usage patterns in the organelle genomes of *Sorbus*, we calculated the RSCU values for protein-coding genes in 263 chloroplast and six mitochondrial genomes. RSCU values exhibited minimal variation among the 263 chloroplast genomes (Figure 6a). Most codons with RSCU values greater than 1 ended in A or T, including TTA (leucine), GTT and GTA (valine), ACT (threonine), TAT (tyrosine), GAT (aspartic acid), and AGA (arginine). Overall, *Sorbus* chloroplast genomes displayed a preference for A/T-ending codons. The six mitochondrial genomes also exhibited variations in synonymous codon usage (Figure 6b). Several A/T-ending codons, such as AGA and CGA (arginine), CTT, TTA, and TTG (leucine), TCA and TCT (serine), ACT (threonine), and GTT (valine), had RSCU values greater than 1. In contrast, codons including GCG, CGC, CGG, CTG, and ACG had RSCU values lower than 1. Both chloroplast and mitochondrial genomes demonstrated a preference for A/T-ending synonymous codons.

Predicted RNA-editing sites showed both common and species-specific patterns among the six mitochondrial genomes (Figure 6c). Relatively high numbers of predicted editing sites were consistently detected in several genes, including *ccmB*, *ccmC*, *ccmFN*, *nad1*, *nad4*, *nad5*, and *nad7*. Among these genes, *nad4* contained the highest number of predicted editing sites in most species. Clear interspecific differences were observed in other genes. *S. alnifolia* had more predicted editing sites in *cox3* than the other species, whereas *S. aucuparia* showed a marked increase in *matR*. The number of editing sites in *nad3* was higher in *S. discolor* and *S. bristoliensis*, while *S. bristoliensis* showed fewer editing sites in *nad4* than the other species.

**Figure 7:**
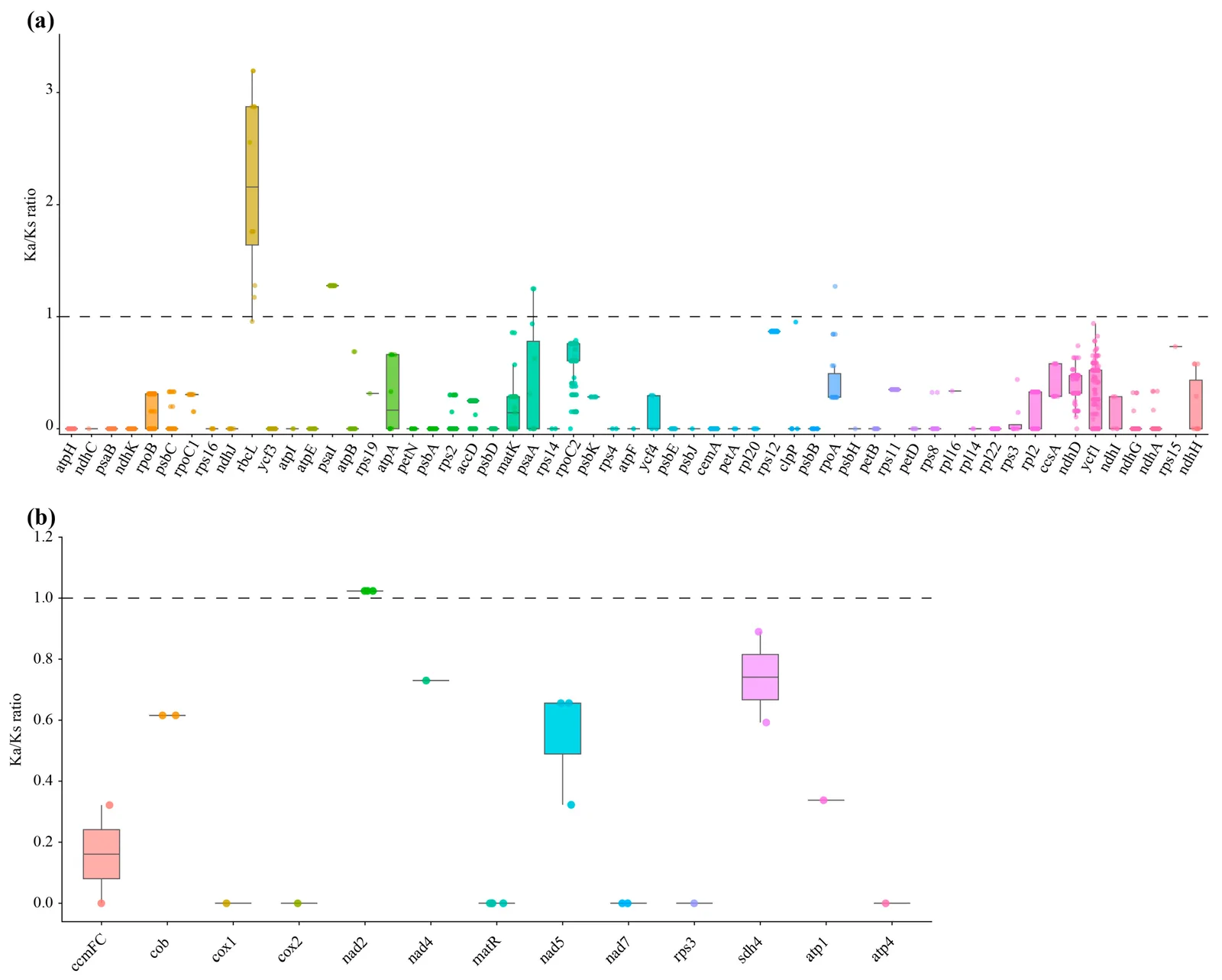
Selective pressures on protein-coding genes in chloroplast and mitochondrial genomes of *Sorbus*. (**a**) Gene-wise Ka/Ks ratios for shared chloroplast protein-coding genes. (**b**) Gene-wise Ka/Ks ratios for mitochondrial protein-coding genes with calculable estimates. The horizontal dashed line indicates Ka/Ks = 1.

To evaluate selective pressures on chloroplast and mitochondrial genes in *Sorbus*, we calculated the Ka/Ks ratios of shared protein-coding genes (Figure 7). Comparisons yielding Ks = 0 or undefined Ka/Ks values, due to the absence of synonymous or nonsynonymous substitutions, were excluded from further analysis. For chloroplast genes (Figure 7a), the majority of genes had Ka/Ks ratios below 1, indicating that most chloroplast genes were predominantly under purifying selection. However, elevated Ka/Ks ratios (>1) were observed in limited comparisons for several genes, including *rbcL*, *psaI*, *psaA*, and *rpoA*. Mitochondrial protein-coding genes exhibited a narrower range of Ka/Ks variation (Figure 7b). Most mitochondrial genes had Ka/Ks ratios below 1, consistent with purifying selection. Only *nad2* showed a Ka/Ks value slightly above 1, whereas *sdh4*, *nad4*, *nad5*, and *cob* exhibited relatively high ratios that remained below this threshold. In contrast, several genes, including *cox1*, *cox2*, *matR*, *rps3*, and *atp4*, showed values close to zero. Overall, purifying selection predominated in both chloroplast and mitochondrial genomes, although the strength of selective pressure varied among genes.

### 2.7. Plastid-Derived Sequences in Mitochondrial Genomes

Plastid-derived homologous sequences were identified in all six mitochondrial genomes (Supplementary Table S8). After length filtering and manual removal of redundant hits, a total of 35 non-redundant regions were retained, including 4 in *S. discolor*, 7 in *S. pohuashanensis*, and 6 each in *S. aucuparia*, *S. alnifolia*, *S. bristoliensis*, and *S. torminalis*. Several plastid loci were detected in all six species, including *rps19*, *ycf1*, *psbF*, *psbD*, and *petG*, whereas *psaB* was detected in *S. aucuparia*, *S. alnifolia*, *S. bristoliensis*, and *S. torminalis*. Most retained regions were located in mitochondrial intergenic regions, while others occurred within *rps4*, *rrn5*, and several mitochondrial tRNA genes.

### 2.8. Phylogenetic Analysis

**Figure 8:**
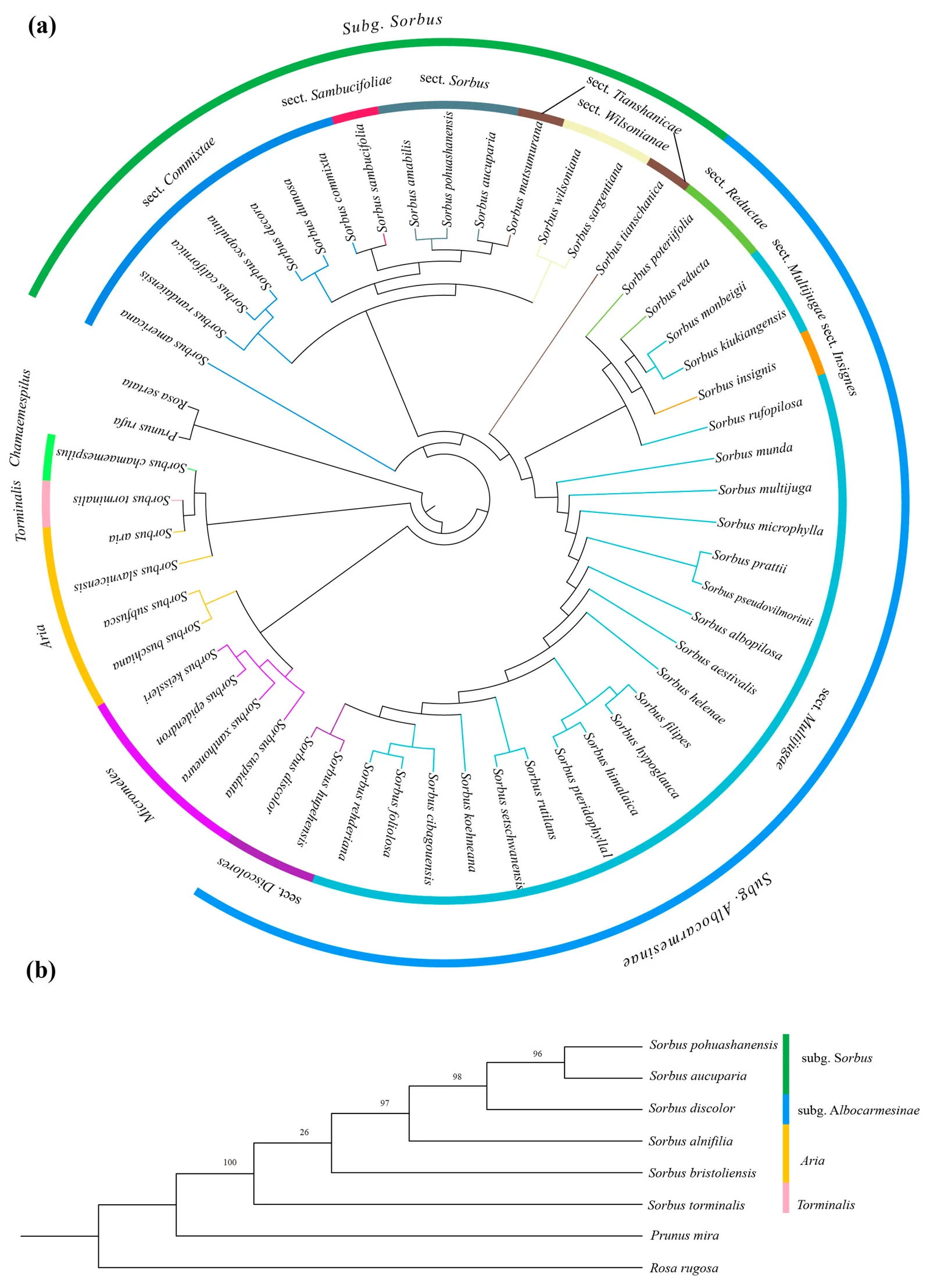
Chloroplast and mitochondrial phylogeny of *Sorbus*. (**a**) Chloroplast phylogeny of *Sorbus* species. The maximum-likelihood tree was reconstructed in IQ-TREE from the concatenated alignment of shared chloroplast protein-coding genes, with *R. sertata* and *P. rufa* as outgroups. Branch labels indicate support from 1000 ultrafast bootstrap replicates. Colored annotations indicate taxonomic affiliations. (**b**) Mitochondrial phylogeny of *Sorbus* species. The maximum-likelihood tree was reconstructed in IQ-TREE from the concatenated alignment of shared mitochondrial protein-coding genes, with *R. rugosa* and *P. mira* as outgroups. Branch labels indicate support from 1000 ultrafast bootstrap replicates. Branch lengths are not drawn to scale.

The final chloroplast and mitochondrial supermatrices comprised 68 shared protein-coding genes (57,322 bp) and 28 shared protein-coding genes (26,677 bp), respectively. Based on the chloroplast supermatrix, the ML tree of 51 *Sorbus* s. l. species exhibited generally strong bootstrap support (Figure 8a and Figure S2a). All compound-leaved species clustered within a well-supported *Sorbus* s. s. clade, which divided into two major lineages corresponding to subg. *Sorbus* and subg. *Albocarmesinae*. The subg. *Sorbus* lineage primarily consisted of sect. *Commixtae*, sect. *Sambucifoliae*, sect. *Sorbus*, sect. *Tianshanicae*, and sect. *Wilsonianae*, whereas sect. *Reductae*, sect. *Multijugae*, sect. *Insignes*, and sect. *Discolores* were assigned to subg. *Albocarmesinae*. These two major lineages aligned with the classification of McAllister [8]. In contrast, among the simple-leaved species, *Aria* was divided into two separate lineages. One lineage was clustered with *Micromeles*, whereas the other was clustered with *Chamaemespilus* and *Torminalis*. Within the latter lineage, *Torminalis* was most closely related to *Aria*. Thus, *Aria* was not recovered as monophyletic in the chloroplast tree. The mitochondrial ML tree of six *Sorbus* species showed a similar pattern (Figure 8b and Figure S2b). The three compound-leaved species, *S. pohuashanensis*, *S. aucuparia*, and *S. discolor*, formed a well-supported clade. The three simple-leaved species did not cluster together and were positioned outside the compound-leaved clade, with *S. torminalis* occupying the basal position among the six *Sorbus* species. Thus, among the sampled taxa, both chloroplast and mitochondrial phylogenies exhibited broadly similar patterns, with the sampled compound-leaved species clustering together and the sampled simple-leaved species occurring closer to the base of the trees.

## 3. Discussion

### 3.1. Genome Structure and Repetitive Elements of Chloroplast and Mitochondrial Genomes

The chloroplast and mitochondrial genomes of *Sorbus* exhibited contrasting patterns of genome evolution. The 263 chloroplast genomes analyzed in this study were highly conserved in structure. Across these genomes, genome size, GC content, and gene composition varied within narrow ranges, consistent with previous plastome comparisons in *Sorbus* [36,37]. At the intraspecific level, the chloroplast genomes of *S. discolor* and *S. pohuashanensis* showed relatively minor variation in genome size and GC content, alongside identical gene composition, supporting structural conservation within each species. Although changes in IR boundaries can contribute to plastome size variation, small shifts in IR/SC junctions generally have only minor effects on overall plastome size [19]. In *Sorbus*, the IR regions showed only slight variation in length, with only minor differences among species. The structural conservation of chloroplast genomes is also commonly observed in other closely related species [38,39,40,41], reflecting the relatively stable evolutionary pattern of chloroplast genomes. 

In contrast, the six *Sorbus* mitochondrial genomes showed greater variation in genome size. The newly assembled mitochondrial genome of *S. discolor* was smaller than the previously reported *Sorbus* mitochondrial genomes and contained the lowest numbers of SSRs and LSRs, whereas *S. alnifolia*, which had the largest mitochondrial genome, contained the greatest numbers of both repeat types. These differences suggest that variation in repetitive sequences may partly contribute to mitochondrial genome size variation among the six sampled species. In contrast to genome size, mitochondrial GC content remained relatively stable at 45.20–45.39%. Similar patterns of variable mitochondrial genome size and relatively stable GC content have been reported in other plants, including *Hippophae* [32] and *Camellia* [30]. Whole-genome comparisons revealed substantial structural differences among the six *Sorbus* mitochondrial genomes, including regions classified as inversions, translocations, and duplications, consistent with the greater structural dynamism reported in other plant mitogenomes [30,33]. Because plant mitochondrial genomes may have alternative structural configurations, these differences should be interpreted as variations among the analyzed assemblies rather than as all possible in vivo conformations.

Repeat sequences were abundant in both organelle genomes. Chloroplast SSRs were dominated by A/T-rich mononucleotide motifs and were mainly located in non-coding regions, a pattern commonly reported in plant plastomes. These characteristics are consistent with previous plastome studies of *Sorbus* [36] and other plants [42], suggesting that non-coding regions represent major hotspots for repeat accumulation and sequence divergence. Mitochondrial SSRs were also mainly located in intergenic regions, and forward and palindromic repeats were the major LSR types, although their numbers varied considerably among species. Repetitive sequences can provide substrates for homologous recombination and may contribute to structural rearrangements in plant mitochondrial genomes. However, because recombination was not directly tested in the present study, the contribution of these repeats to mitochondrial genome rearrangement in *Sorbus* remains to be confirmed.

### 3.2. Plastome Sequence Variation Across Interspecific and Intraspecific Scales

At the sequence level, the chloroplast dataset showed a different pattern from the strong structural conservation described above. Genome-wide diversity across the interspecific dataset is best interpreted as sequence diversity among the sampled *Sorbus* taxa rather than as population-level diversity. Within species, both *S. discolor* and *S. pohuashanensis* showed high haplotype diversity, with *S. pohuashanensis* showing higher Hd, Pi, and k values than *S. discolor*. Importantly, this qualitative difference remained after one IR copy was removed before whole-plastome diversity calculations, indicating that the higher plastome diversity observed in *S. pohuashanensis* was not an artifact of double weighting the duplicated IR sequence. The distribution of nucleotide diversity differed between *S. discolor* and *S. pohuashanensis*, and the most variable genes and intergenic regions identified in the two species were not identical. Previous *Sorbus* plastome studies identified candidate hypervariable regions mainly from interspecific comparisons [36,37], whereas the present dense intraspecific sampling shows that regions ranked as highly variable among taxa are not necessarily the most informative within a species. These results suggest that highly variable regions identified from a limited number of individuals representing different species may not necessarily be the most informative regions within a particular species. Extensive intraspecific plastome sampling may therefore be more useful for identifying candidate regions for within-species studies and marker development [20,21,22,23,24]. Notably, *trnH*-*GUG*–*psbA* showed relatively high nucleotide diversity at both the interspecific and intraspecific levels, suggesting that it may be a useful candidate region for marker development, although its performance should be further tested before wider use.

### 3.3. Organelle Gene Evolution and Mitochondrial Sequence Variation

Most chloroplast and mitochondrial protein-coding genes of *Sorbus* had Ka/Ks ratios below 1, indicating that purifying selection predominated during chloroplast and mitochondrial genome evolution in *Sorbus*. In the chloroplast genomes, *rbcL* exhibited relatively high Ka/Ks ratios in several chloroplast comparisons, whereas *psaI*, *psaA*, and *rpoA* also showed elevated values in a subset of comparisons. Previous studies have reported signatures of positive selection or elevated evolutionary rates in *rbcL* in several plant lineages [43,44,45]. However, pairwise Ka/Ks estimates can be strongly affected by low synonymous divergence, particularly when closely related sequences are compared. Among mitochondrial genes, only *nad2* showed a Ka/Ks value slightly above 1. Elevated Ka/Ks ratios for *nad2* have also been reported in *Chimonanthus praecox* [46] and *Coptis* species [47], indicating that this gene may experience different selective pressures among plant lineages. Further analyses using broader taxon sampling and codon-based evolutionary models will be required to determine whether these genes have experienced lineage-specific positive selection.

A particularly notable pattern was the contrasting evolutionary state of mitochondrial *sdh3* among the six sampled species. An intact coding sequence was retained in *S. alnifolia*, *S. bristoliensis*, and *S. torminalis*, whereas *S. discolor* retained a homologous region containing a premature stop codon, and no intact mitochondrial *sdh3* was detected in *S. pohuashanensis* or *S. aucuparia*. Previous studies have shown that *sdh3* and *sdh4* are among the mitochondrial genes that are relatively prone to loss and transfer to the nucleus in angiosperms, and closely related lineages may therefore exhibit different states of retention, pseudogenization, and loss [48,49]. Interestingly, *S. discolor* retained both a putative nuclear *sdh3* homolog and a pseudogenized mitochondrial *sdh3*, whereas *S. pohuashanensis* contained a putative nuclear *sdh3* homolog, with no intact mitochondrial *sdh3* detected. The coexistence of different *sdh3* states among closely related *Sorbus* species therefore suggests that degeneration and loss of this gene have occurred unevenly during lineage divergence. Putative nuclear *sdh3* homologs were identified in both *S. discolor* and *S. pohuashanensis*, indicating that nuclear homologous sequences are present even in species with different mitochondrial *sdh3* states. A similar coexistence of a mitochondrial *sdh3* pseudogene and a nuclear *sdh3*-related sequence has also been reported in Rosaceae species [50,51], suggesting that this pattern may not be unique within Rosaceae. However, sequence similarity alone cannot establish a specific mitochondrial-to-nuclear transfer event, functional replacement, or the evolutionary timing of these changes.

Other mitochondrial sequence features further illustrate the complexity of organelle genome evolution among the six sampled species. Predicted RNA-editing sites were concentrated mainly in nad and ccm genes, consistent with patterns reported in many other plant mitogenomes [33,52]. The number of predicted RNA-editing sites may be influenced by differences in gene content and CDS length and requires further experimental validation. Additionally, plastid-derived homologous regions were detected in all six sampled mitochondrial genomes, consistent with the widespread exchange of homologous sequence between plastid and mitochondrial genomes in plants [53]. The presence of both shared and species-specific plastid-derived homologous regions indicates variation in mitochondrial sequence composition among the sampled species, although their transfer direction and timing cannot be determined from sequence similarity alone.

### 3.4. Organelle Phylogenetic Implications and Limitations

The plastome phylogeny recovered all sampled compound-leaved species within *Sorbus* s. s., which was further divided into two major lineages corresponding to subg. *Sorbus* and subg. *Albocarmesinae*, consistent with the classification of McAllister [8]. In contrast, the simple-leaved groups were placed outside *Sorbus* s. s., consistent with previous molecular studies supporting the separation of *Sorbus* s. s. from lineages such as *Aria*, *Chamaemespilus*, and *Torminalis* [35]. Notably, *Aria* was not recovered as monophyletic in the plastome tree, with *Chamaemespilus* and *Torminalis* nested within one *Aria* lineage, consistent with the plastome-based phylogeny reported by Hu et al. [54]. This pattern further highlights the unresolved relationships among the simple-leaved lineages of *Sorbus* s. l. The mitochondrial phylogeny, although based on only six *Sorbus* species, showed a broadly similar pattern, with the three sampled compound-leaved species forming a clade and the sampled simple-leaved species positioned outside it. However, the narrower mitochondrial taxon sampling limits the resolution of relationships among the major lineages, and the mitochondrial tree should therefore be regarded as complementary evidence rather than an independent reconstruction of the evolutionary history of *Sorbus*.

Interpretation of organelle phylogenies in *Sorbus* is further complicated by the frequent occurrence of hybridization, polyploidization, and apomixis [13,14,15,16,17]. These processes can result in discordance between cytoplasmic and nuclear genealogies, particularly when hybridization is followed by organelle capture. Consequently, chloroplast and mitochondrial trees primarily reflect the evolutionary histories of the sampled organelle genomes and may not fully represent species relationships. Earlier studies proposed that compound-leaved *Sorbus* s. s. may have originated from simple-leaved ancestors [55,56], and the placement of the sampled simple-leaved lineages outside *Sorbus* s. s. is compatible with this hypothesis. A similar change in leaf type has been proposed in *Acer*, where simple-leaved lineages were located near the base of the phylogeny and compound leaves were suggested to have evolved later [57]. However, the direction of leaf-form evolution cannot be established from organelle phylogenies alone. Broader mitochondrial sampling, nuclear phylogenetic analyses, and explicit ancestral-state reconstruction will be necessary to test this hypothesis and to clarify the evolutionary relationships among the major lineages of *Sorbus*.

## 4. Materials and Methods

### 4.1. Plant Materials, Genome Assembly, and Annotation

We de novo assembled 71 complete *S. discolor* chloroplast genomes and 143 complete *S. pohuashanensis* chloroplast genomes from previously generated Illumina whole-genome resequencing data deposited in the National Genomics Data Center (NGDC) (CRA044970). The individuals originated from multiple localities within the natural ranges of the two species. Detailed sampling information, including locality, longitude, latitude, and elevation, is summarized in Table S9. Raw-read quality was assessed using FastQC v0.11.5 (https://www.bioinformatics.babraham.ac.uk/projects/fastqc/, accessed on 19 September 2026), and chloroplast genomes were independently assembled using GetOrganelle v1.7.1 [58] with default parameters. Assembly graphs were inspected in Bandage v0.8.1 [59], and genome orientations and IR boundaries were checked and standardized using CPStools v3.0 [60], with manual correction in Geneious v11.1.5 [61], when necessary. The quality of the 214 newly assembled plastomes was evaluated using two approaches. First, average base coverage was obtained from GetOrganelle assembly logs. Second, raw reads were mapped back to the assembled plastomes using minimap2 v2.26-r1175 [62], and genome coverage and mean read depth were calculated with SAMtools v1.21 [63]. Final sequences were further checked in Geneious, and only complete assemblies without gaps or ambiguous bases were retained. The complete mitochondrial genome of *S. discolor* was assembled from previously generated PacBio HiFi reads deposited under CRA044972 using HiMT v1.1.3 [64]. Raw PacBio HiFi reads were mapped back to the assembled mitochondrial genome using minimap2 and read-mapping coverage and sequencing depth were calculated using SAMtools.

Chloroplast genome annotation was performed using CPGAVAS2 (http://47.96.249.172:16019/analyzer/home, accessed on 7 April 2025) [65] with published *S. discolor* (NC037696) and *S. pohuashanensis* (ON478195) chloroplast genomes as references. The *S. discolor* mitochondrial genome was annotated using the Plant Mitochondrial Genome Annotator (PMGA; http://www.1kmpg.cn/pmga/, accessed on 24 August 2025) [66] with the 319-mitochondrial genome database, followed by manual inspection. The rRNA genes were identified by BLASTn v2.16.0 [67] searches and tRNA annotations were validated using tRNAscan-SE v2.0.11 [68]. In addition, 49 published complete chloroplast genomes and 5 mitochondrial genomes of *Sorbus* were downloaded from NCBI GenBank (Supplementary Tables S10 and S11), resulting in a final dataset of 263 chloroplast genomes and 6 mitochondrial genomes.

### 4.2. Genome Characteristics and Comparative Analyses of Organelle Genomes

The total length, number of unique genes, and GC content of each chloroplast and mitochondrial genome were calculated using a custom Python script implemented in Python v3.12.10, together with the lengths and GC contents of the large single-copy (LSC), small single-copy (SSC), and inverted-repeat (IR) regions. To assess structural conservation and variation in IR expansion and contraction, the junctions of the LSC, SSC, IRa, and IRb regions were compared among the chloroplast genomes of 51 species using CPStools v3.0. For mitochondrial genome comparisons, pairwise whole-genome alignments among the six representative species were performed using MUMmer v4.0.1 [69]. Collinear blocks and structural rearrangements were identified using SyRI v1.7.1 [70] and visualized using plotsr v1.1.1 [71]. Structural comparisons were based on the final mitochondrial genome assemblies, and alternative repeat-mediated configurations were not independently reconstructed.

Simple sequence repeats (SSRs) and long sequence repeats (LSRs) were characterized in both chloroplast and mitochondrial genomes. SSRs were identified using MISA v2.1 [72], with minimum repeat thresholds of 10, 6, 4, 4, 3, and 3 repeat units for mono-, di-, tri-, tetra-, penta-, and hexanucleotide motifs, respectively. Long sequence repeats were detected using REPuter web server (https://bibiserv.cebitec.uni-bielefeld.de/reputer/, accessed on 13 September 2025) [73] and classified into four categories: forward (F), reverse (R), palindromic (P), and complement (C) repeats, with a minimum repeat length of 30 bp.

### 4.3. Mitochondrial sdh3 Variation and Identification of Putative Nuclear Homologs

Previous comparisons of mitochondrial gene content revealed differences in the presence and integrity of *sdh3* among the examined species. The intact mitochondrial *sdh3* sequence of *S. torminalis* was used as a reference to identify homologous regions in the other mitogenomes. Candidate sequences were extracted from GenBank files using Biopython v1.88 [74], and aligned using MAFFT v 7.526 [75] with the --auto strategy to evaluate sequence integrity and identify internal stop codons. Because *S. discolor* and *S. pohuashanensis* showed mitochondrial *sdh3* degeneration or apparent absence, respectively, their nuclear genomes were further examined for potential *sdh3* homologs. The genome assembly and gene annotation of *S. discolor* (GWHJNAC00000000) and *S. pohuashanensis* (GWHBDNT00000000) were obtained from the Genome Warehouse. Candidate nuclear proteins were identified by sequence similarity searches and validated using BLASTp online tool (NCBI; https://blast.ncbi.nlm.nih.gov/Blast.cgi?PAGE=Proteins, accessed on 25 September 2025) against the NCBI protein database. Corresponding genomic regions, CDSs, predicted proteins, and exon–intron structures were extracted from the genome and annotation files, and gene structures were visualized using TBtools v2.485 [76].

### 4.4. Nucleotide Diversity Analysis

To avoid double weighting of duplicated IR sequences, one IR copy was removed from each plastome before whole-plastome sequence-diversity analyses. All 263 complete chloroplast genome sequences were aligned using MAFFT v7.526, and sites containing gaps or missing data were excluded from diversity analyses. DnaSP v6 [77] was used to calculate the number of segregating sites (S), total number of mutations (Eta), number of haplotypes (h), haplotype diversity (Hd), nucleotide diversity (Pi), and the average number of pairwise nucleotide differences (k). These diversity statistics were calculated for the complete dataset of sampled *Sorbus* taxa and separately for *S. discolor* (71) and *S. pohuashanensis* (143). The geographic distributions of plastome haplotypes in *S. discolor* and *S. pohuashanensis* were visualized according to sampling coordinates using the ggplot2 v4.0.3 [78] and scatterpie v0.2.6 [79] packages in R v4.5.0.

To identify highly variable chloroplast genomic regions, shared genes and intergenic regions were extracted using CPStools v3.0 for the complete *Sorbus* dataset, *S. discolor*, and *S. pohuashanensis*, respectively. Each region was aligned using MAFFT v7.526, and nucleotide diversity (Pi) was calculated using DnaSP v6. For mitochondrial genomes, shared protein-coding sequences (CDSs) among the six representative species were extracted using PhyloSuite v1.2.3 [80]. Each shared CDS was aligned individually using MAFFT v7.526 with default parameters, and nucleotide diversity (Pi) was calculated using DnaSP v6.

### 4.5. Codon Usage and Mitochondrial RNA-Editing Analyses

Shared protein-coding sequences were extracted from the 263 chloroplast genomes and six representative mitochondrial genomes and concatenated for each genome. Relative synonymous codon usage (RSCU) values were calculated using CodonW v1.4.2 [81] to evaluate codon usage patterns. Stop codons were retained in the RSCU calculation. Potential RNA-editing sites in mitochondrial protein-coding genes were predicted using the Deepred-Mt module [82] implemented in the PMGA. A cut-off value of 0.5 was used for prediction.

### 4.6. Selective Pressure Analyses

The shared protein-coding genes extracted from the chloroplast and mitochondrial genomes were translated into their corresponding amino acid sequences using PhyloSuite v1.2.3. Codon-based alignments were generated using ParaAT v2.0 [83], and nonsynonymous substitution rates (Ka), synonymous substitution rates (Ks), and Ka/Ks ratios were subsequently calculated using KaKs_Calculator 3.0 [84]. *S. discolor* accession SD0001 and *S. discolor* were used as the reference sequences for the chloroplast and mitochondrial datasets, respectively. The Ka/Ks ratios were used to assess gene-specific selective pressures, with values <1 indicating predominant purifying selection, values close to 1 indicating approximately neutral evolution, and values >1 suggesting potential positive selection. Comparisons with Ks = 0 were excluded from further analysis.

### 4.7. Identification of Plastid-Derived Mitochondrial Sequences

To identify potential plastid-derived sequences in mitochondrial genomes, the BLASTn online tool (NCBI; https://blast.ncbi.nlm.nih.gov/Blast.cgi?PAGE=Nucleotides, accessed on 5 October 2025) was used to perform pairwise sequence comparisons between the mitochondrial genome and corresponding chloroplast genomes of each of the six representative species. Homologous fragments were retained using a minimum sequence identity of 70% and an E-value threshold of ≤1 × 10^−5^, and only matches with a minimum length of ≥100 bp were retained as putative plastid-derived mitochondrial sequences. Redundant hits from overlapping or duplicated plastid regions were manually removed.

### 4.8. Phylogenetic Analyses

Phylogenetic relationships were reconstructed using shared protein-coding sequences (CDSs) from the chloroplast and mitochondrial genomes. For the chloroplast phylogeny, shared protein-coding genes were extracted from 51 species representing the major lineages of *Sorbus*, with *R. sertata* (MT506347) and *Prunus rufa* (PV624663) designated as outgroups. For the mitochondrial phylogeny, shared protein-coding genes were extracted from the six representative species with *R. rugosa* (ON478175) and *P. mira* (NC065231) as outgroups.

Shared protein-coding genes were aligned individually using MAFFT v7.526 and trimmed with trimAl v1.5.1 [85] using the -automated1 option, and the resulting alignments were concatenated into unpartitioned nucleotide supermatrices. Maximum likelihood (ML) phylogenetic trees were reconstructed in IQ-TREE2 v2.0.7 [86], with the best-fitting substitution models selected by ModelFinder [87]. GTR+F+I+R4 and GTR+F+R2 were selected for the chloroplast and mitochondrial datasets, respectively. Branch support was assessed using 1000 ultrafast bootstrap replicates.

## 5. Conclusions

This study presents an integrated perspective on organelle genome evolution in *Sorbus* by combining extensive intraspecific plastome sampling with comparative analysis of six mitochondrial genomes. The sampled plastomes were structurally conserved, while substantial sequence variation was detected at both interspecific and intraspecific levels. Notably, the distinct nucleotide diversity patterns observed in *S. discolor* and *S. pohuashanensis* underscore the necessity of extensive intraspecific sampling for identifying informative variable regions and assessing genetic diversity. In contrast, mitochondrial genomes displayed greater structural dynamism, characterized by significant variations in genome size and frequent rearrangements, thereby highlighting the divergent evolutionary trajectories of these two organelle genomes. Overall, these findings provide valuable comparative organelle genome resources for elucidating genomic variation in *Sorbus*, facilitating future marker development and genetic resource studies.

## Figures and Tables

**Table 1:** Comparative characteristics of newly assembled chloroplast genomes.

Characteristic	*S. discolor* (*n* = 71)	*S. pohuashanensis* (*n* = 143)
Total length (bp)	160,005–160,197	159,869–160,206
LSC length (bp)	87,944–88,158	87,759–88,167
IR length (bp)	26,378–26,410	26,378–26,416
SSC length (bp)	19,258–19,342	19,261–19,387
Total GC (%)	36.51–36.55	36.51–36.57
LSC GC (%)	34.21–34.27	34.21–34.31
SSC GC (%)	30.24–30.31	30.20–30.32
IR GC (%)	42.64–42.66	42.63–42.65
Unique genes	112	112
Protein-coding genes	78	78
rRNA genes	4	4
tRNA genes	30	30
Multi-copy gene number	17	17
One-intron genes	15	15
Two-intron genes	3	3

**Table 2:** General features of six mitochondrial genomes in *Sorbus*.

Species	Genome Size (bp)	GC (%)	Unique Genes	Protein-Coding Genes	tRNAs	rRNAs
*S. discolor*	372,987	45.36	56	34	19	3
*S. pohuashanensis*	396,857	45.36	53	33	17	3
*S. aucuparia*	384,977	45.39	53	33	18	2
*S. alnifolia*	455,361	45.20	55	34	17	4
*S. bristoliensis*	386,757	45.31	55	34	18	3
*S. torminalis*	386,758	45.31	53	33	17	3

**Table 3:** Genome-wide plastome sequence diversity at interspecific and intraspecific levels in the sampled *Sorbus* taxa.

Level	*n*	S	Eta	h	Hd	Pi	k
Interspecific	263	8560	8730	99	0.970	0.00130	164.602
Intraspecific (*S. discolor*)	71	316	320	19	0.873	0.00025	32.963
Intraspecific (*S. pohuashanensis*)	143	359	365	44	0.959	0.00049	64.378

## Data Availability

All complete chloroplast genomes and mitochondrial genome newly assembled in the present study have been deposited in the National Center for Biotechnology Information (NCBI) GenBank database. The accession numbers for the 214 newly assembled chloroplast genomes are PZ573387, PZ573474–PZ573650, and PZ573652–PZ573687. The accession number of mitochondrial genome of *S. discolor* is PZ771142.

## Supplementary Materials

The following supporting information can be downloaded at: https://www.mdpi.com/article/10.3390/ijms27209324/s1.

## Abbreviations

The following abbreviations are used in this manuscript:

NGDC	National Genomics Data Center
NCBI	National Center for Biotechnology Information
LSC	Large single-copy
SSC	Small single-copy
IR	Inverted repeat
PMGA	Plant Mitochondrial Genome Annotator
SSR	Simple sequence repeat
LSR	Long sequence repeat
CDS	Coding sequence
Hd	Haplotype diversity
Pi	Nucleotide diversity
RSCU	Relative synonymous codon usage
Ka	Nonsynonymous substitution rate
Ks	Synonymous substitution rate
ML	Maximum likelihood
