# Supplementary material for: Comparative Chloroplast and Mitochondrial Genomics of Sorbus L. Sensu Lato: Genome Diversity, Structural Variation, and Phylogeny
Source: Int J Mol Sci. 2026 Oct 20;27(20):9324. doi: 10.3390/ijms27209324 (PMC13705202; doi:10.3390/ijms27209324)
Supplement: Supplementary file 1 [file ijms-27-09324-s001.zip › Supplementary Figure.pdf]

Supplementary Figure

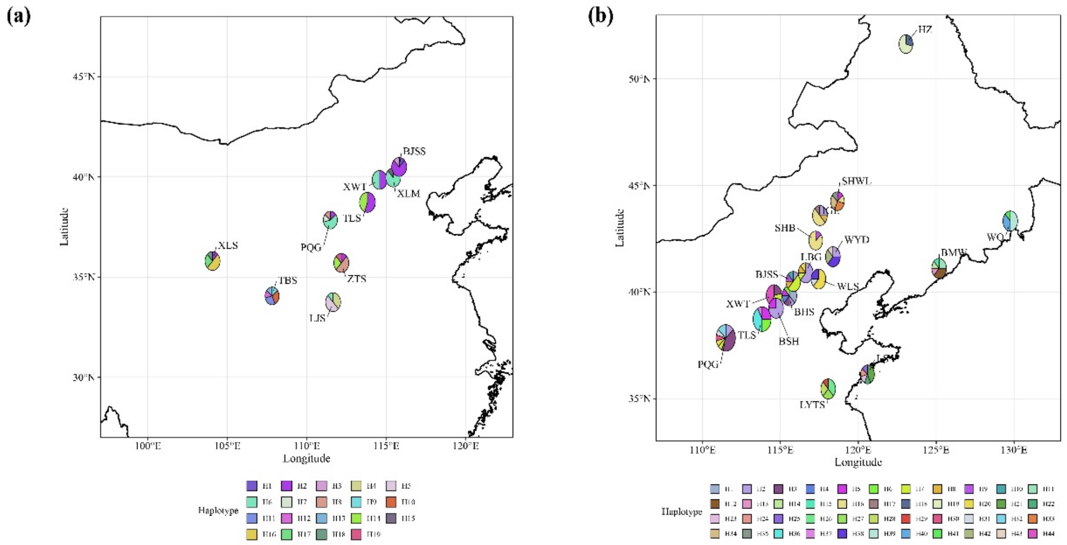

Figure S1. Geographic distribution of plastome haplotypes in *S. discolor* and *S. pohuashanensis*. (a) Geographic distribution of the 19 plastome haplotypes identified in *S. discolor*. (b) Geographic distribution of the 44 plastome haplotypes identified in *S. pohuashanensis*. Pie charts indicate haplotype composition at each sampling locality, with different colors representing different haplotypes. Pie-chart size is proportional to the number of sampled individuals at each locality.

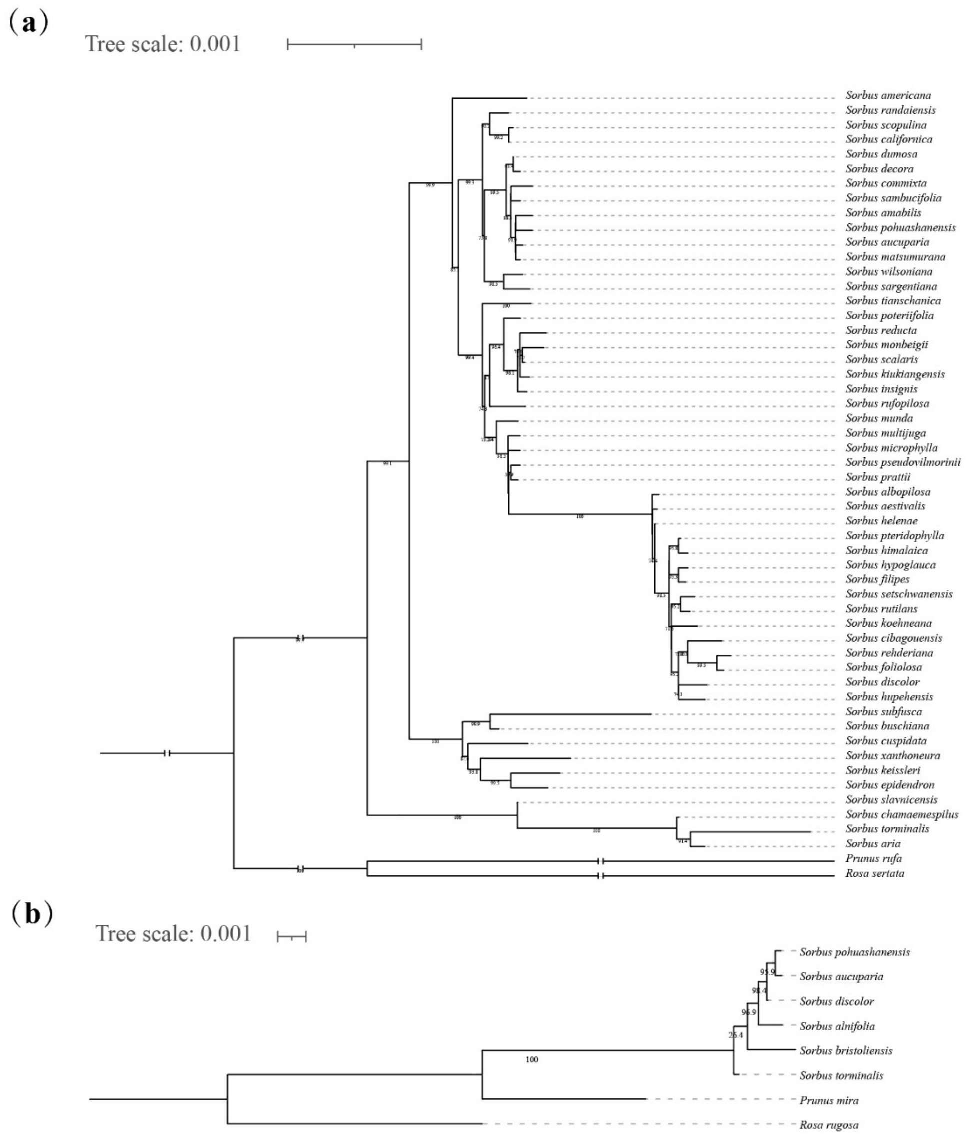

Figure S2. Maximum-likelihood phylograms of *Sorbus* based on chloroplast and mitochondrial protein-coding genes. (a) Chloroplast phylogram based on shared protein-coding genes. (b) Mitochondrial phylogram based on shared protein-coding genes. Branch lengths are proportional to substitutions per site, and numbers at nodes indicate bootstrap support values. Break marks on branches indicate that the corresponding branch lengths were truncated for visualization.
